# A Novel Cytotoxic Steroidal Saponin from the Roots of *Asparagus cochinchinensis*

**DOI:** 10.3390/plants10102067

**Published:** 2021-09-30

**Authors:** Ji-Young Kim, He Yun Choi, Hye Mi Kim, Jung-Hye Choi, Dae Sik Jang

**Affiliations:** 1 Department of Biomedical and Pharmaceutical Sciences, Graduate School, Kyung Hee University, Seoul 02447, Korea; k_christina@khu.ac.kr; 2 Department of Life and Nanopharmaceutical Sciences, Graduate School, Kyung Hee University, Seoul 02447, Korea; choiheyun@khu.ac.kr; 3 College of Pharmacy, Kyung Hee University, Seoul 02447, Korea; hyemi586@gmail.com

**Keywords:** *Asparagus cochinchinensis*, cytotoxicity, Liliaceae, ovarian cancer cells, steroidal saponin

## Abstract

A new steroidal saponin, 26-*O*-*β*-d-glucopyranosyl-(25*R*)-furost-5-*ene*-3*β*,22*α*,26-triol 3-*O*-(1−4)-*β*-d-glucopyranosyl-*α*-l-rhamnopyranosyl-(1−2)-[*α*-l-rhamnopyranosyl-(1−4)]-*β*-d-glucopyranoside [asparacochioside A (**1**)] was isolated from a hot water extract of the roots of *Asparagus cochinchinensis*, together with the known steroidal saponins protodioscin (**2**), methyl protodioscin (**3**), aspacochioside A (**4**), aspacochioside C (**5**), 15−hydroxypseudoprotodioscin (**6**), and chamaedroside E (**7**). The structure of the new compound **1** was determined by interpretation of its spectroscopic data (1D- and 2D-NMR and HR−Q−TOF−MS) and sugar analysis. The isolated compounds **1**−**7** were tested for their in vitro cytotoxicity against human ovarian cancer cell lines (A2780 and SKOV3). Asparacochioside A (**1**) exhibited a significant cytotoxicity against both A2780 and SKOV3 cells with IC50 values of 5.25 ± 2.2 and 46.82 ± 9.43 μM, respectively. Furthermore, asparacochioside A (**1**) significantly increased the percentage of Annexin V-positive cells (apoptotic cells), suggesting that asparacochioside A induces ovarian cancer cell death via apoptosis.

## 1. Introduction

The genus *Asparagus* (Liliaceae) comprises over three hundred species distributed around the world and used in salads and as herbs and vegetables. *Asparagus cochinchinensis* (Loureio) Merrill is a perennial herb distributed in Eastern Asia, including many provinces of China, Japan, and Korea [1]. The dried roots of *A. cochinchinensis* have been used in Traditional Chinese Medicine for more than two thousand years to treat fevers, renal failure, heart diseases, and lung cancer [2].

Moreover, extensive chemical studies on the roots of *A. cochinchinensis* have led to the identification of many compounds, such as steroidal saponins, mono-, oligo-, and polysaccharides, and amino acids [3,4]. The extracts of *A. cochinchinensis* and the corresponding constituents have been shown to have several pharmacological effects, including anti-oxidant [5] and anti-inflammatory effects [6]. Additionally, cytotoxicity against several human cancer cell lines such as cervical cancer HeLa, lung cancer NCI−H460, breast cancer MCF-7, and liver cancer HepG2 cells has been reported [5,7]. Steroidal saponins from *A. cochinchinensis* and other plants such as *Smilax davidiana* and *Polygonautm sibiricum* are also known to have anti−inflammatory effects by inhibiting cytokine production and cytotoxicity against tumor cell lines [8,9].

In our continuing search for bioactive compounds from medicinal plants, the roots of *A. cochinchinensis* were chosen for a phytochemical investigation, since its hot water extract was found to have significant cytotoxicity against human ovarian carcinoma (A2780) and human female ovarian adenocarcinoma (SKOV3) cell lines with 30 and 39% cell growth inhibition at 200 μg/mL, respectively.

In the present study one new (compound **1**) and six known steroidal saponins **2**−**7** were purified from the roots of *A. cochinchinensis* by repeated chromatography. The structure of the new compound **1** was elucidated by interpreting 1D- and 2D-nuclear magnetic resonance (NMR) spectroscopic data analysis, acid hydrolysis, and high-resolution quadrupole time of flight mass (HR−Q−TOF−MS) spectrometric data analysis. All the isolated compounds **1**−**7** were evaluated for their cytotoxicity against ovarian cancer cell lines (A2780 and SKOV3). To summarize, in this paper, we describe the isolation of steroidal saponins **1**−**7** from the roots of *A. cochinchinensis*, structure elucidation of the new steroidal saponin **1**, and cytotoxicity of the isolates against human ovarian cancer cells.

## 2. Results and Discussion

### 2.1. Structure Elucidation of Compound ***1***

**Figure 1:**
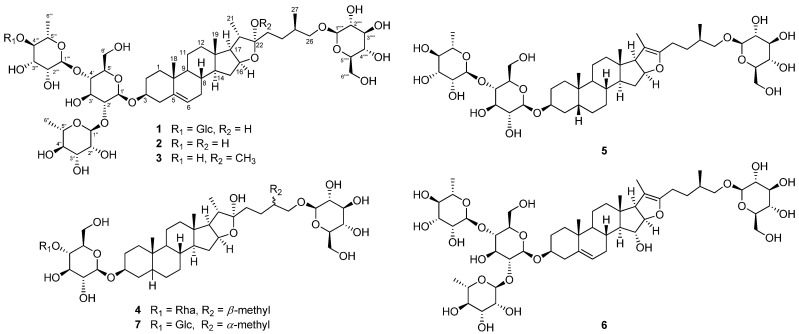
Structures of compounds **1**–**7** isolated from the roots of *A. cochinchiensis*.

One new (compound **1**) and six known furostane-type steroidal saponins **2**−**7** were isolated from the roots of *A. cochinchinensis* (Figure 1).

Compound **1** was isolated as a pale brown powder. The molecular formula was established as C_57_H_94_O_27_ through HR−Q−TOF−MS isotopic ion peaks at *m*/*z* = 1209.5892 [M − H]^−^ (calcd for C_57_H_93_O_27_, 1209.5904) (Figure S1). The ^1^H-NMR spectrum of **1** exhibited six methyl signals at *δ*_H_ 0.89 (3H, s), 0.99 (3H, d, *J* = 6.5 Hz), 1.08 (3H, s), 1.34 (3H, d, *J* = 7.0 Hz), 1.67 (3H, d, *J* = 6.0 Hz), 1.77 (3H, d, *J* = 6.0 Hz), and five anomeric protons at *δ*_H_ 4.81 (1H, d, *J* = 7.5 Hz), 4.95 (1H, d, *J* = 7.5 Hz), 5.24 (1H, d, *J* = 7.5 Hz), 5.87 (1H, s), and 6.42 (1H, s) (Table 1, Figure S2) [10]. The ^13^C-NMR, distoriionless enhancement by polarization transfer (DEPT)135-NMR, and ^1^H-^13^C heteronuclear single quantum coherence spectroscopy (HSQC) spectra showed 57 signals with six methyl signals at *δ*_C_ 16.4 × 2, 17.4, 18.3, 18.6, and 19.3, 13 methylene carbon signals at *δ*_C_ 21.0, 28.3, 30.1, 32.2, 32.4, 37.1, 37.4, 38.9, 39.8, 78.5, 61.1, 62.3, and 62.7, nine methine carbons in aglycone moiety including an olefinic carbon at *δ*_C_ 31.6, 34.2, 40.6, 50.3, 56.6, 63.7, 78.0, 81.0, and 121.8, and four quaternary carbons at *δ*_C_ 37.1, 40.7, 110.6, and 140.7 (Table 1, Figures S3 and S4). According to the ^1^H- and ^13^C-NMR and HSQC data, compound **1** could be expected to have five sugars [*δ*_C_/*δ*_H_ 100.3/4.95, 1H, d (7.5); 102.0/5.88, 1H, s; 101.9/6.42, 1H, s; 106.7/5.24, 1H, d (7.5); 104.8/4.81, 1H, d (7.5)]. Based on the ^1^H-^1^H correlation spectroscopy (COSY) and ^1^H-^13^C heteronuclear multiple bond correlation (HMBC) data (Figures S5 and S6), it was inferred that compound **1** is a furostane-type steroidal saponin.

**Figure 2:**
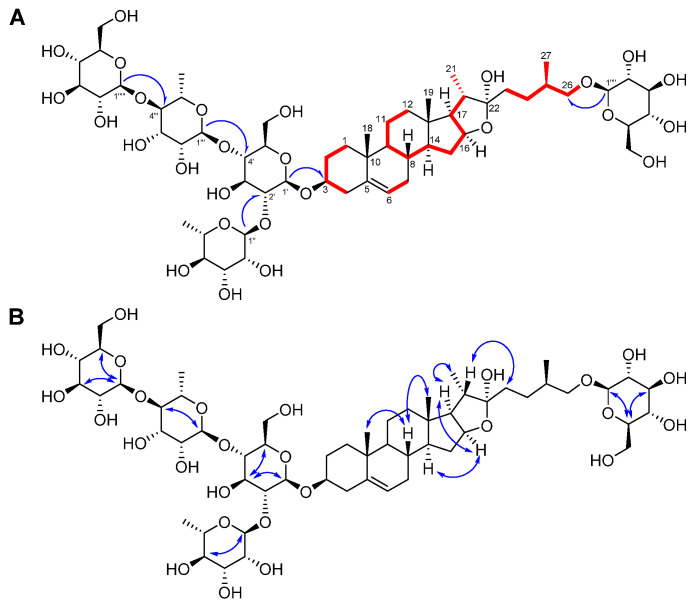
Key ^1^H−^1^H COSY (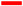) and HMBC (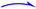) correlations of compound **1** (A). Key ^1^H−^1^H NOESY (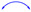: blue dashed arrows) correlations of compound **1** (B).

Through analysis of the HMBC and nuclear Overhauser effect correlation spectroscopy (NOESY) spectra (Figure 2, Figures S6 and S7), it was proposed that compound **1** has three glucoses and two rhamnoses [11]. The positions of the sugars were determined by analysis of the HMBC correlations; H−Glc−1′ [δ 4.95 (1H, d, *J* = 7.5 Hz)] with C−3 (δ 78.0), H−Rha−1′’ [δ 6.42 (1H, s)] with C−Glc−2′ (δ 78.0), H−Rha−1′’’ [δ 5.87 (1H, s)] with C−Glc−4′ (*δ* 78.4), and H−Glc−1′’’’’ [*δ* 5.24 (1H, d, *J* = 7.5 Hz)] with C−26 (*δ* 78.5) (Figure 2). The glucoses and rhamnoses in compound **1** were identified as *β*-d- and *α*-l-forms, respectively, based on the coupling constants of the anomeric protons and acid hydrolysis followed by high performance liquid chromatography (HPLC) analysis. The ^1^H- and ^13^C-NMR spectroscopic data of compound **1** were very similar to those of protodioscin (**2**), except for the presence of an additional *β*-glucose unit at Rha-4′’’ in **1** (Table 2 and Table 3, Figure 1) [10]. The chemical structure of compound **1** was very resemble to costucoside I, isolated from the seeds of *Costus speciosus* which only differ from the position of Glc−1′’’’at Rha−3′’’ [12]. The absolute configuration of the methyl group at C-25 was determined as *R* by the difference in proton NMR chemical shifts between H_2_−26*α* and H_2_−26*β* (∆_ab_ = 0.32) [13,14]. Thus, the chemical structure of the new furostane-type steroidal saponin **1** was elucidated as 26-*O*-*β*-d-glucopyranosyl-(25*R*)-furost-5-*ene*-3*β*,22*α*,26-triol 3-*O*-(1−4)-*β*-d-glucopyranosyl-*α*-l-rhamnopyranosyl-(1−2)-[*α*-l-rhamnopyranosyl-(1−4)]-*β*-d-glucopyranoside and named asparacochioside A.

The other known compounds were identified as protodioscin (**2**) [10], methyl protodioscin (**3**) [15], aspacochioside A (**4**) [16], aspacochioside C (**5**) [16], 15-hydroxypseudoprotodioscin (**6**) [17], and chamaedroside E (**7**) [18] by comparison of their NMR spectroscopic data with those previously reported data.

### 2.2. The Cytotoxicity of the Compounds Isolated from A. cochinchinensis against Human Ovarian Cancer Cells

To evaluate the potential anti-tumor activity of the isolates from the hot water extract of *A. cochinchinensis*, the cytotoxicity of compounds **1**−**7** against human ovarian cancer cells (A2780 and SKOV3) were examined using a 3-[4,5-dimethylthiazol-2-yl]-2,5-diphenyl tetrazolium bromide (MTT) assay.

**Figure 3:**
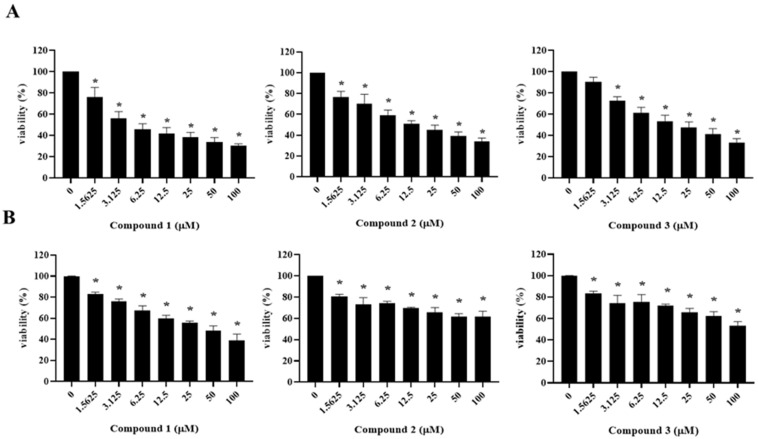
The effects of compounds **1**−**3** on cell viability in human ovarian cancer cells A2780 (**A**) and SKOV3 (**B**). Ovarian cancer cells (A2780 and SKOV3) were treated with compounds **1**−**3** in the indicated concentrations for 48 h. Data are presented as mean ± SD and evaluated by one-way ANOVA analysis. * *p* < 0.05 as compared with the untreated group.

As shown in Table 3 and Figure 3, asparacochioside A (**1**), protodioscin (**2**), and methyl protodioscin (**3**) exhibited a significant cytotoxicity in A2780 cells with IC_50_ values of 5.25, 10.14, and 21.78 μM, respectively, in a dose dependent manner. In SKOV3 cells, only the new compound asparacochioside A (**1**) showed a significant cytotoxicity with observed IC_50_ value of 46.82 μM, while compounds **4**−**7** were not active against either A2780 or SKOV3 human ovarian cancer cells (>100 μM). Overall, among the seven compounds isolated from *A. cochinchinensis*, only the novel compound **1** showed a potent cytotoxicity against both A2780 and SKOV3, suggesting its potential anti−cancer activity in human ovarian cancer.

Both protodioscin (**2**) and methyl protodioscin (**3**) have been reported to show various biological activities such as anti-tumor and anti-inflammatory effects [19,20]. However, this is the first study to demonstrate their cytotoxicity against human ovarian cancer cells. Protodioscin (**2**) showed more potent cytotoxicity against A2780 cells than methyl protodioscin (**3**), suggesting that the methylation at C-22 hydroxy group may interrupt the activity of protodioscin (**3**). Although asapacochioside C (**5**) was cytotoxic against human lung adenocarcinoma A549 cells [21], it failed to show a significant cytotoxicity against human ovarian cancer cells in this study. Reportedly, chamaedroside E (**7**) was first isolated from *Veronica chamaedrys* [18]. However, the presence of **7** in *A. cochinchinensis* was found in this study, for the first time.

### 2.3. Induction of Apoptotic Cell Death by Asparacochioside A *(**1**)* in Human Ovarian Cancer Cells

**Figure 4:**
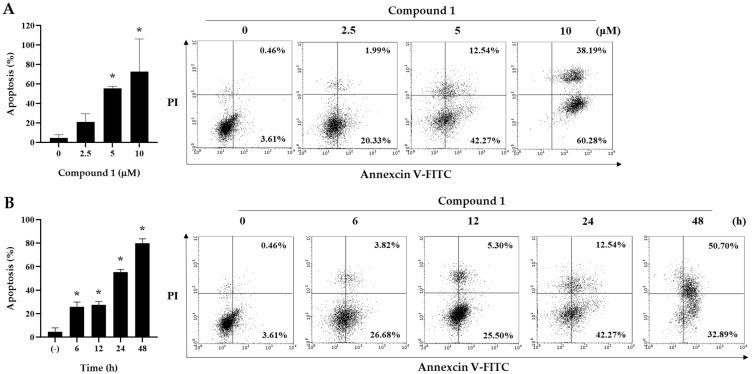
The effect of compound **1** on apoptosis in A2780 cells. Cells were treated with compound **1** for the indicated concentrations (2.5, 5.0, and 10 μM) (**A**) and times (6, 12, 24, and 48 h) (**B**), and then co-stained with propidium iodide (PI) and Annexin V-FITC. The graph indicates the percentage of apoptosis. Data are presented as mean ± SD and evaluated by one-way ANOVA analysis. * *p* < 0.05 as compared with control.

To further investigate whether the cytotoxicity of asparacochioside A (**1**) was associated with the induction of apoptosis, Annexin V-FITC staining was performed. Asparacochioside A (**1**) significantly increased the percentage of Annexin V positive cells (apoptotic cells) in a dose- and time-dependent manner (Figure 4). These results indicate that asparacochioside A-induced cytotoxicity was associated with apoptosis in human ovarian cancer cells.

## 3. Materials and Methods

### 3.1. Plant Material

The roots of *Asparagus cochinchinensis* (Loureio) Merrill (Liliaceae) were purchased from Nanuum pharmaceutical Co. (Youngcheon-si, Kyungsangbukdo, South Korea), in May 2017. The plant material was identified by D.S.J. and a voucher specimen (ASCO1-2017) has been deposited in the Lab. of Natural Product Medicine, Kyung Hee University.

### 3.2. General Experimental Procedures

General experimental procedures are provided in the Supplementary Materials.

### 3.3. Isolation of Compounds ***1**−**7***

Dried roots of *A. cochinchinensis* (800 g) were extracted with distilled water (8 L) at 100 °C for 2 h, and the solvent was removed using rotary evaporator. The extract (300.0 g) was subjected to Diaion HP column chromatography (CC, 9.6 × 56.0 cm), eluting with MeOH−H_2_O gradient system (from 0:1 to 1:0 *v*/*v*) to afford 12 fractions (C1~C12). Fraction C4 (3.8 g) was separated by Sephadex LH-20 CC (4.3 × 57.5 cm) with acetone−H_2_O (4:6 *v*/*v*) to make four fractions (C4−1~C4−4). Fraction C4−3 (2.2 g) was subjected further to silica gel CC (3.3 × 34.0 cm, 230−400 mesh) with EtOAc−acetone−H_2_O mixture (3:6:1 *v*/*v*/*v*), yielding seven subfractions (C4−3−1~C4−3−7). Chamaedroside E (**7**, 21.5 mg) was purified by HPLC with Gemini 5 µm NX-C18 110A column (acetonitirle–H_2_O = 25:75 to 40:60, *v*/*v*) from subfraction C4−3−4 (60.8 mg). Fraction C6 (12.0 g) was loaded to a silica gel column (230−400 mesh; 5.6 × 35.0 cm) and eluted with CH_2_Cl_2_−MeOH−H_2_O solvent system [14:6:1 *v*/*v*/*v*] to afford seven subfractions (C6−1~C6−7). Subfraction C6−4 (1.75 g) was subjected to silica gel CC (5.2 × 25.5 cm, 230−400 mesh) with EtOAc−acetone−H_2_O mixture (from 4:5:1 to 0:8:2 *v*/*v*/*v*), yielding protodioscin (**2**, 217.8 mg). Subfraction C6−5 (7.16 g) was subjected to silica gel CC (230−400 mesh; 5.2 × 32.0 cm) eluting with EtOAc−acetone−H_2_O [4:5:1 to 3.5:5:1.5 *v*/*v*/*v*] and afforded methylprotodioscin (**3**, 1.56 g). Aspacochioside A (**4**, 118.7 mg) was separated from subfraction C6−2 (392.0 mg) by silica gel CC (3.6 × 26.0 cm, 230−400 mesh) with EtOAc−acetone−H_2_O mixture (4:5:1 *v*/*v*/*v*). Subfraction C6−6 (2.11 g) was fractionated by silica gel CC (230−400 mesh; 4.4 × 30.0 cm), eluting with EtOAc−acetone−H_2_O gradient system [from 4:5:1 to 3.5:5:1.5 *v*/*v*/*v*] to yield compound **1** (724.2 mg).

Fraction C7 (16.3 g) was subjected to silica gel CC (5.8 × 40.0 cm, 230−400 mesh) with EtOAc−acetone−H_2_O mixture (4:5:1 *v*/*v*/*v*) to generate nine subfractions (C7−1~C7−9). 15−Hydroxy−pseudoprotodioscin (**6**, 211.0 mg) was purified by flash chromatography using a Redi Sep−C18 cartridge (130 g, acetonitrile−H_2_O, from 15:85 to 40:60 *v*/*v*) from C7−6 (1.6 g). Protodioscin (**2**, 2.08 g was obtained additionally from subfraction C7−8 (9.7 g) by flash chromatography using a Redi Sep−C18 cartridge (130 g, acetonitrile−H_2_O, from 15:85 to 45:55 *v*/*v*).

Fraction C9 (406.6 mg) was fractionated further by flash chromatography using a Redi Sep−C18 cartridge (130 g, acetonitrile−H_2_O, from 10:90 to 40:60 *v*/*v*) to afford aspacochioside C (**5**, 68.6 mg).

#### Asparacochioside A (**1**)

Pale brown powder; m.p.: 188.2 °C; ${\left[\alpha \right]}_{D}^{20}$: −63° (*c* 0.1, MeOH); IR (ATR) νmax 3410, 1745, 1712 cm^−1^; HR−Q−TOF−MS (negative mode) *m*/*z* = 1209.5892 [M − H]^−^, (calcd for C57H93O27, 1209.5904); ^1^H- and ^13^C-NMR data, see Table 1 and Table 2.

### 3.4. Acidic Hydrolysis of Compound ***1***

Compound **1** (10.0 mg) was hydrolyzed with 1N HCl at 80 °C for 3 h. Sodium bicarbonate was added to stop the reaction.

### 3.5. Absolute Configurations of the β-Glucose and α-Rhamnose in Compound ***1***

The absolute configurations of *β*-glucose and α-rhamnose in **1** was determined using the method reported by Tanaka et al. [21]. The hydrolysate was derivatized with pyridine (500 μL), l-cysteine methyl ester hydrochloride (1.2 mg), and *σ*-tolyl isothiocyanate (100 μL) and analyzed using HPLC. The glucose and rhamnose in the reaction mixture of **1** was detected at 30.7 and 35.5 min, respectively. The retention times of authentic d-glucoses and l-rhamnose were 30.8 and 35.4 min, respectively, under the same HPLC conditions. Therefore, the absolute configuration of β-glucose in **1** was confirmed as the d configuration, whereas α-rhamnose was confirmed as the l configuration.

### 3.6. Cell Culture

A2780 and SKOV3 human ovarian cancer cell lines were obtained from American Type Culture Collection (ATCC). The cells were maintained in Roswell Park Memorial Institute (RPMI) 1640 (Life Technologies Inc., Grand Island, NY, USA) medium with penicillin (100 U/mL), 5% fetal bovine serum (FBS), and streptomycin sulfate (100 μg/mL, Life Technologies Inc.). Cells with medium were kept in humidified atmosphere of 5% CO and 37 °C temperatures.

### 3.7. MTT Assay

The cell growth was assessed using the MTT assay. Briefly, Cells were seeded in a 96-well plate at a density of 1 × 10^5^/mL per well. After 24 h, cells were treated with different concentrations of extract or compounds for 48h. Following incubation, 50 μL of MTT (1 mg/mL stock solution, molecular probes Inc., Eugene, OR, USA) was added and incubated for 4 h in the 37 °C incubator. The medium was discarded and the formazan crystals were dissolved in DMSO. The absorbance was measured using a microplate spectrophotometer (SpectraMax; Molecular Devices, Sunnyvale, CA, USA) at 540 nm.

### 3.8. Annexin V−FITC Staining Assay for Apoptosis Analysis

Apoptotic cells were detected by the binding of fluorescent Annexin V (Annexin V-FITC). Cells were treated with asparacochioside A (**1**) for indicated time periods or concentrations. After incubation, both floating and adherent cells from each well collected in tubes and washed with PBS. The cells were then re-suspended in 500 μL of binding buffer and stained with 2.75 μL of FITC-conjugated Annexin V for 15min in darkness. Finally the stained cells were washed with binding buffer and stained with 5 μL of PI (50 mg/mL) prior to analysis using a Guava^®^ easyCyte flow cytometer (Merck Millipore, Burlington, MA, USA).

### 3.9. Statistical Analysis

All statistical parameters were calculated using GraphPad Prism 5.0 (GraphPad Software, Inc., La Jolla, CA, USA). Data are presented as mean ± SD and evaluated by unpaired Student *t*-test or one-way ANOVA analysis. Difference with a *p*-value less than 0.05 was considered to be statistically significant.

## 4. Conclusions

Seven compounds **1**‒**7**, including a new furostane-type steroidal saponin named asparacochioside A (**1**) were isolated from the roots of *A. cochinchinensis* in the present study. The chemical structure of the new compound was determined using their spectroscopic data (^1^H-NMR, ^13^C-NMR, DEPT, HSQC, HMBC, NOESY, and HR−Q−TOF−MS) measurement and by acidic hydrolysis. Isolation of chamaedroside E (**7**) from the roots of *A. cochinchinensis* was reported for the first time in this paper. Among the isolates, asparacochioside A (**1**), protodioscin (**2**), and methyl protodioscin (**3**) exhibited a significant cytotoxicity against A2780 cells. In SKOV3 cells, only the new compound asparacochioside A (**1**) showed a significant cytotoxicity. Compound **1** induced apoptotic cell death in human ovarian cancer cells and significantly increased the percentage of annexin V positive cells (apoptotic cells) in a dose- and time-dependent manner. Thus, asparacochioside A (**1**) is worthy of additional experiments for its potential as an anti-cancer agent.

## Figures and Tables

**Table 1:** ^1^H- and ^13^C-NMR spectroscopic data for the aglycones of compounds **1** and **2** (*δ* in ppm, C_5_D_5_N, 800 and 200 MHz).

Position	1		Protodioscin (2) *^a^*	
	*δ*_H_ Multi (*J* in Hz)	*δ*_C_	*δ*_H_ Multi (*J* in Hz)	*δ*_C_
1	1.85, 1H, m/2.07, 1H, m	30.1	1.86, 1H, m/2.08, 1H, m	29.8
2	1.44, 2H, m	21.0	1.54, 2H, m	20.9
3	3.88, 1H, d (11.0, 6.5)	78.0	3.81, 1H *^b^*	78.4
4	2.73, 1H, d (11.0)/2.79, 1H, d (10.5)	38.9	2.74, 1H, d (11.5)/2.79, 1H, d (10.5)	38.8
5	-	140.7	-	140.6
6	5.30, 1H, s	121.8	5.34, 1H, s	121.8
7	1.88, 2H, m	32.2	1.86, 2H, m	32.2
8	1.57, 1H, m	31.6	1.56, 1H, m	31.5
9	0.90, 1H *^b^*	50.3	0.90, 1H *^b^*	50.1
10	-	37.1	-	37.1
11	0.98, 1H *^b^*/1.32, 1H *^b^*	37.4	0.97, 1H *^b^*/1.30, 1H *^b^*	37.8
12	1.12, 1H, m/1.74, 1H, m	39.8	1.12, 1H, m/1.75, 1H, m	39.7
13	-	40.7	-	40.6
14	1.07, 1H *^b^*	56.6	1.07, 1H *^b^*	56.4
15	1.48, 1H *^b^* / 2.02, 1H *^b^*	32.4	1.48, 1H, m/2.03, 1H, m	32.3
16	4.96, 1H, t (4.5)	81.0	4.46, 1H, m	81.1
17	1.93, 1H *^b^*	63.7	1.94, 1H *^b^*	63.7
18	1.08. 3H, s	19.3	1.05, 3H, s	19.2
19	0.89, 3H, s	16.4	0.89, 3H, s	16.3
20	2.25, 1H, q (2.0)	40.6	2.24, 1H, m	40.5
21	1.34, 3H, d (7.0)	16.4	1.34, 3H, d (7.0)	16.3
22	-	110.6	-	111.0
23	2.07, 2H *^b^*	37.1	1.57, 2H, dd (10.5, 5.0)	30.0
24	1.68, 1H, d (4.0)/2.05, 1H, m	28.3	1.68, 1H, d (4.0)/2.05, 1H, m	28.2
25	1.94, 1H *^b^*	34.2	1.96, 1H *^b^*	34.1
26	3.63, 1H, dd (9.5, 6.0)/3.95, 1H, dd (9.0, 6.5)	78.5	3.63, 1H, d (4.0)/3.99, 1H, d (6.5)	78.4
27	0.99, 3H, d (6.5)	17.4	0.99, 3H, d (6.0)	17.3

***^a^*** NMR data of compound **2** isolated in this study. ***^b^*** signal is overlapped.

**Table 2:** ^1^H- and ^13^C-NMR spectroscopic data for the sugar moieties of compounds **1** and **2** (*δ* in ppm, C_5_D_5_N, 800 and 200 MHz).

Position	1		Protodioscin (2) *^a^*	
	*δ*_H_ Multi (*J* in Hz)	*δ*_C_	*δ*_H_ Multi (*J* in Hz)	*δ*_C_
3-*O*-Glc-1’	4.95, 1H, d (7.5)	100.3	4.96, 1H, d (7.0)	100.1
2’	3.88, 1H *^b^*	78.0	3.78, 1H *^b^*	77.9
3’	3.63, 1H *^b^*	76.9	3.61, 1H *^b^*	76.8
4’	3.95, 1H *^b^*	78.4	3.92, 1H *^b^*	78.3
5’	4.43, 1H *^b^*	77.3	4.40, 1H *^b^*	77.5
6’	4.06, 1H, t (8.0)/4.20, 1H *^b^*	61.1	4.04, 1H, t (8.0)/4.23, 1H *^b^*	61.1
2’-*O*-Rha-1”	6.42, 1H, s	101.9	6.41, 1H, s	101.9
2”	4.67, 1H *^b^*	71.8	4.61, 1H *^b^*	72.4
3”	4.87, 1H, s	72.5	4.75, 1H, s	72.6
4”	4.37, 1H *^b^*	74.00	4.34, 1H *^b^*	73.8
5”	5.04, 1H *^b^*	69.5	4.95, 1H *^b^*	69.4
6”	1.77, 3H, d (6.0)	18.3	1.76, 3H, d (6.0)	18.4
4’-*O*-Rha-1’”	5.87, 1H, s	102.0	5.87, 1H, s	102.7
2’”	4.64, 1H, dd (9.0, 3.0)	72.4	4.61, 1H, dd (9.0, 3.0)	72.4
3’”	4.65, 1H, d (4.5)	72.8	4.61, 1H, m	72.7
4’”	4.21, 1H *^b^*	74.01	4.61, 1H *^b^*	73.3
5’”	4.96, 1H *^b^*	71.2	4.92, 1H *^b^*	70.2
6’”	1.67, 3H, d (6.0)	18.6	1.64, 3H, d (6.0)	18.5
26-*O*-Glc-1”“	4.81, 1H, d (7.5)	104.8	4.87, 1H, d (7.5)	104.8
2”“	4.14, 1H, t (8.5)	75.1	4.14, 1H, t (8.5)	75.0
3”“	3.84, 1H *^b^*	77.9	3.85, 1H, m	77.8
4”“	4.24, 1H *^b^*	71.6	4.23, 1H *^b^*	71.5
5”“	4.29, 1H, t (9.5)	78.5	4.30, 1H, t (9.0)	78.4
6”“	4.36, 1H *^b^*/4.40, 1H *^b^*	62.7	4.36, 1H *^b^*/4.41, 1H *^b^*	62.6
4’”-*O*-Glc-1”“‘	5.24, 1H, d (7.5)	106.7		
2”“‘	4.03, 1H, m	76.5		
3”“‘	3.63, 1H, m/3.95, 1H, m	75.3		
4”“‘	4.23, 1H *^b^*	78.5		
5”“‘	4.30, 1H, t (9.0)	71.2		
6”“‘	4.37, 1H *^b^* 4.51/1H, d (11.5)	62.3		

***^a^*** NMR data of compound **2** isolated in this study. ***^b^*** signal is overlapped.

**Table 3:** The cytotoxicity of compounds **1**−**3** isolated from *A. cochinchinensis* in A2780 and SKOV3 human ovarian cancer cells.

Compound	IC_50_ (*μ*M) *^a^*	
	A2780	SKOV3
**1**	5.25 ± 2.2^*d,e,f*^	46.82 ± 9.43 *^f^*
**2**	10.14 ± 0.12^*c, e*^	>100
**3**	21.78 ± 8.14 *^c,d,e^*	>100
**Cisplatin *^b^***	10.82 ± 0.43 *^c,e^*	17.55 ± 4.46^*c*^

***^a^*** IC_50_ is defined as the concentration that reduces cell number by 50% compared with control cultures. The data represents the means ± SD of the results from three independent experiments. Data are evaluated by two-tailed unpaired *t*-test. ***^b^*** Cisplatin was used as a positive control. ***^c^*** *p* < 0.05 as compared with the compound 1-treated group in the same cells. ***^d^*** *p* < 0.05 as compared with the compound 2-treated group in the same cells. ***^e^*** *p* < 0.05 as compared with the compound 3-treated group in the same cells. ***^f^*** *p* < 0.05 as compared with the cisplatin-treated group in the same cells.

## Supplementary Materials

The following are available online at https://www.mdpi.com/article/10.3390/plants10102067/s1, General Experimental Procedure, The HR-ESI-MS, ^1^H-NMR (800 MHz, C_5_D_5_N), ^13^C-NMR (200 MHz, C_5_D_5_N), DEPT135-NMR, HSQC, COSY, HMBC, and NOESY spectra of **1** (Figures S1–S7).
