# Supplementary material for: A Novel Cytotoxic Steroidal Saponin from the Roots of Asparagus cochinchinensis
Source: Plants (Basel). 2021 Sep 30;10(10):2067. doi: 10.3390/plants10102067 (PMC8538803; doi:10.3390/plants10102067)

plants

MDPI

Supplementary metarials

A Novel Cytotoxic Steroidal Saponin from the Roots of *Asparagus cochinchinensis*

Contents

General Experimental procedure

Figure S1. HR-ESI-MS spectrum of compound **1**

Figure S2. The ^{1}H-NMR (800 MHz, C_5D_5N) spectrum of compound **1**

Figure S3. The ^{13}C- and DEPT135-NMR (200 MHz, C_5D_5N) spectra of compound **1**

Figure S4. The HSQC spectrum of compound **1** in C_5D_5N

Figure S5. The COSY spectrum of compound **1** in C_5D_5N

Figure S6. The HMBC spectrum of compound **1** in C_5D_5N

Figure S7. The NOESY spectrum of compound **1** in C_5D_5N

General Experimental procedure

UV spectra were evaluated on Optizen pop (Mecasys, Daejeon, Korea). MPA 100 (Stanford research systems, Sunnyvale, CA, USA) was used to measure melting points in open capillary tubes. Optical rotations were obtained on a Jasco P-2000 polarimeter (JASCO, Tokyo, Japan), using a 10-cm microcell. AVNACE III HD (Bruker, German) 800 MHz was used for obtaining NMR spectra. HR-Mass spectra were obtained by a Q-TOF micro mass spectrometer (Waters, Milford, Massachusetts, USA). TLC analyses were performed on Silica gel 60 F_{254} (Merck, Kenilworth, MA, USA) and RP-18 F_{254S} (Merck) plates. Compounds were visualized by dipping plates into 20% (v/v) H_2SO_4 reagent (Samchun, Seoul, Korea) and then heated at 110°C for 5-10 min. Agilent Cary 630 FTIR (Agilent Technologies, Santa Clara, CA, USA) was applied to obtain IR spectrum. Sephadex LH-20 (Amersham Pharmacia Biotech, Buckinghamshire, United Kingdom), Silica gel (Merck 60A, 230-400 mesh ASTM), Diaion HP-20 (Mitsubishi, Tokyo, Japan), and reversed-phase silica gel (YMC Co., ODS-A 12 nm S-150 μm) were used for column chromatography. Pre-packed cartridges (Teledyne Isco, Superior St, Lincoln, USA) were used for flash chromatography. Flash chromatography was performed using the flash purification system (Combi Flash Rf, Teledyne Isco). HPLC was performed using Waters purification system (Waters, Milford, Massachusetts, USA) (1525 pump, PDA 1996 detector) with Gemini NX-C18 110A column (250 × 21.2mm i.d. 5 μm, Phenomenex, Torrance, CA, USA Before chromatographic separations, all solvents used for this study were distilled.

Figure S1. HR-ESI-MS spectrum of compound **1**

Single Mass Analysis
Tolerance = 5.0 mDa / DBE: min = -1.5, max = 50.0
Element prediction: Off
Number of isotope peaks used for i-FIT = 3
Monoisotopic Mass, Even Electron Ions
622 formula(e) evaluated with 4 results within limits (up to 50 closest results for each mass)
Elements Used:

Mass	Calc. Mass	mDa	PPM	DBE	Formula	i-FIT	i-FIT Norm	Fit Conf %	C	H	O
1209.5892	1209.5881	1.1	0.9	42.5	C82 H81 O9	280.8	1.023	35.95	82	81	9
	1209.5904	-1.2	-1.0	11.5	C57 H93 O27	280.9	1.158	31.42	57	93	27
	1209.5939	-4.7	-3.9	33.5	C75 H85 O14	281.3	1.495	22.43	75	85	14
	1209.5845	4.7	3.9	20.5	C64 H89 O22	282.1	2.283	10.20	64	89	22

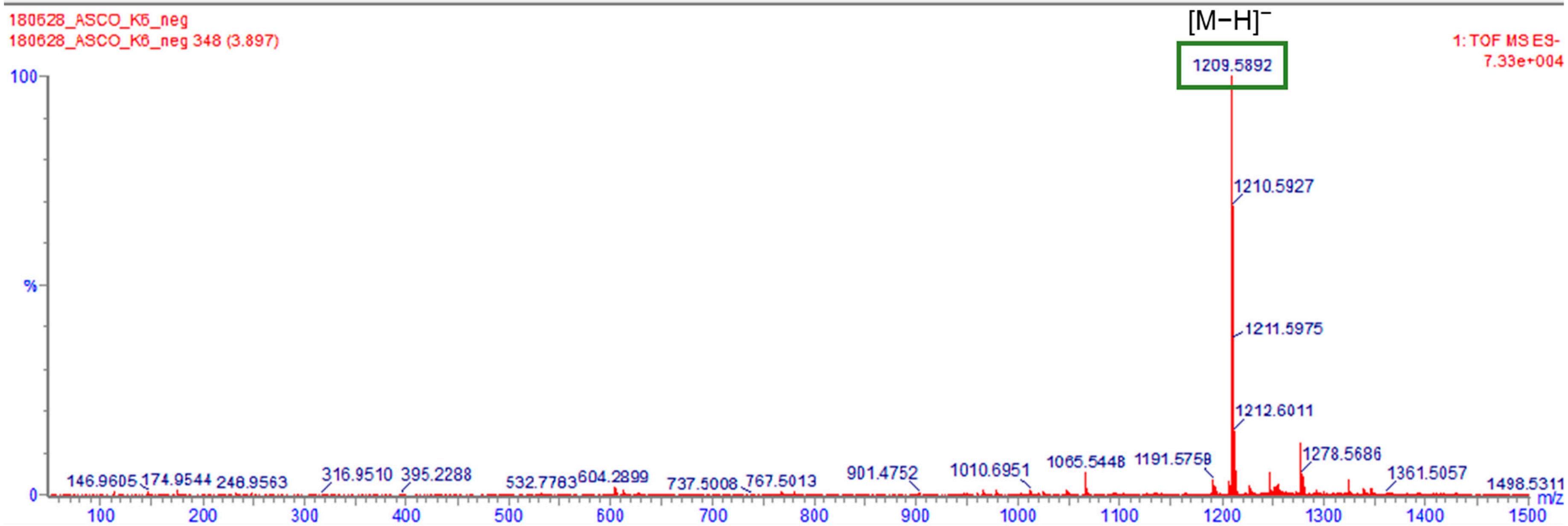

Figure S2. The ^{1}H-NMR (800 MHz, C_5D_5N) spectrum of compound **1**

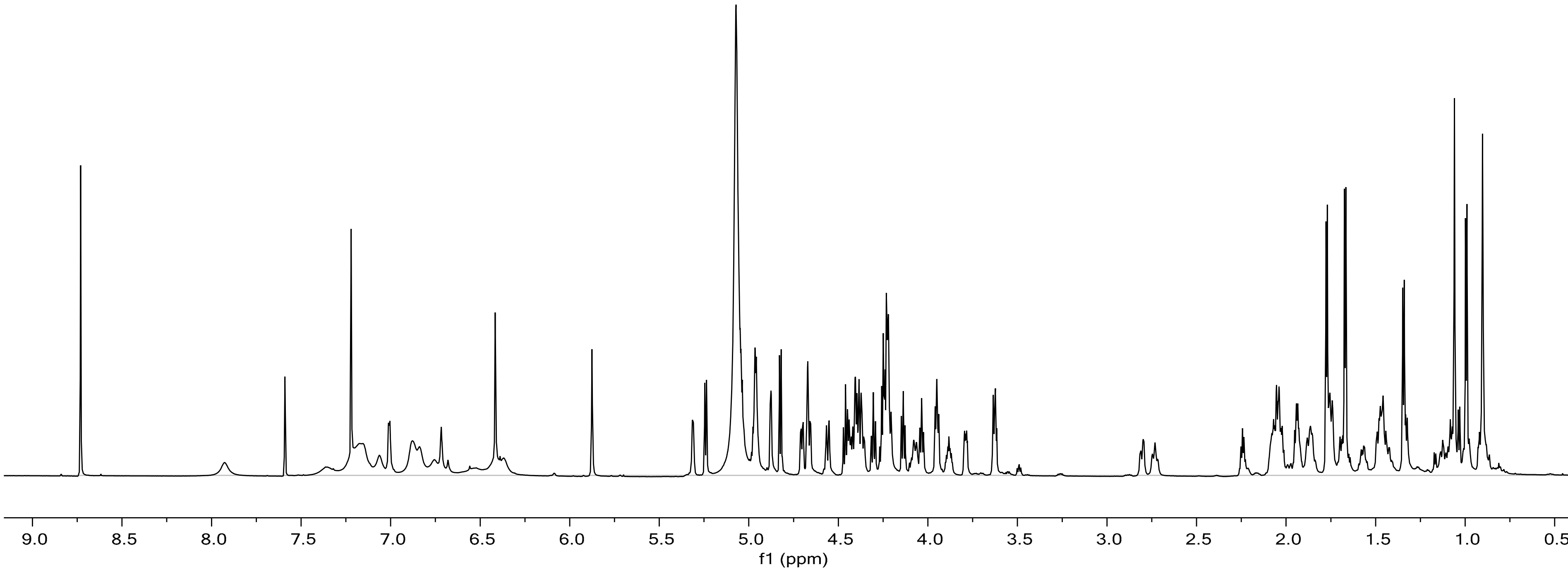

Figure S3. The ^{13}C- and DEPT135-NMR (200 MHz, C_5D_5N) spectra of compound **1**

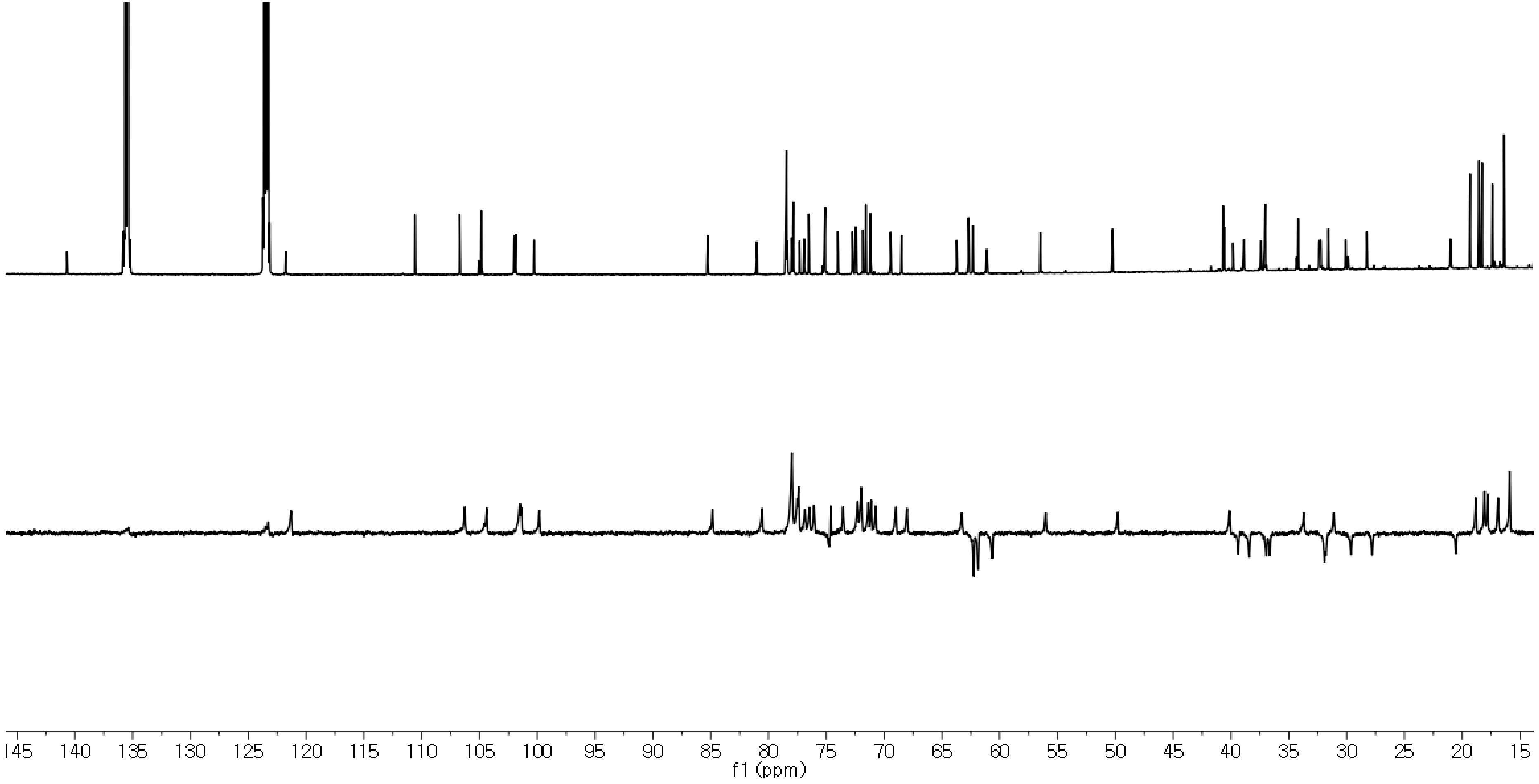

Figure S4. The HSQC spectrum of compound **1** in C_5D_5N

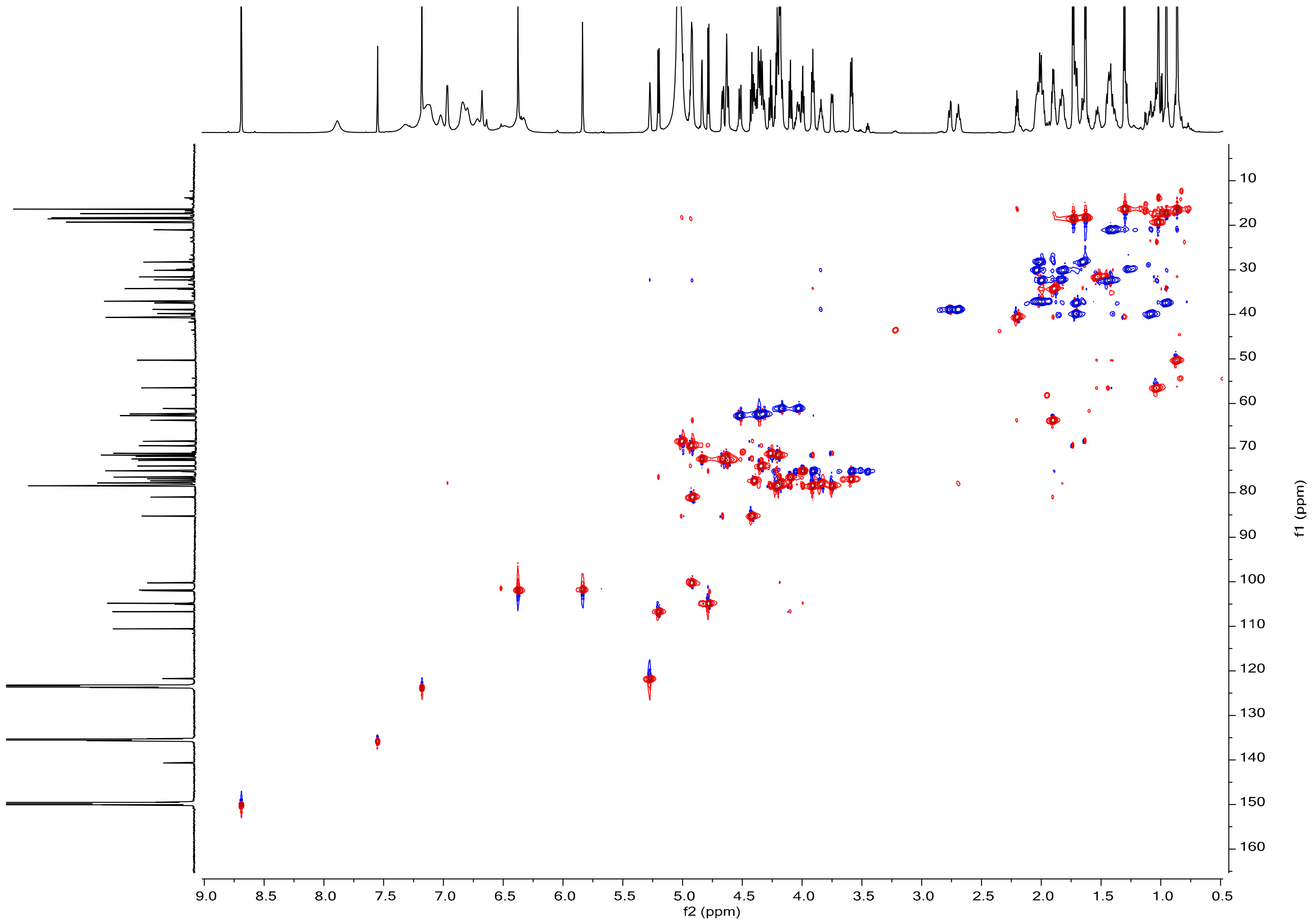

Figure S5. The COSY spectrum of compound **1** in C_5D_5N

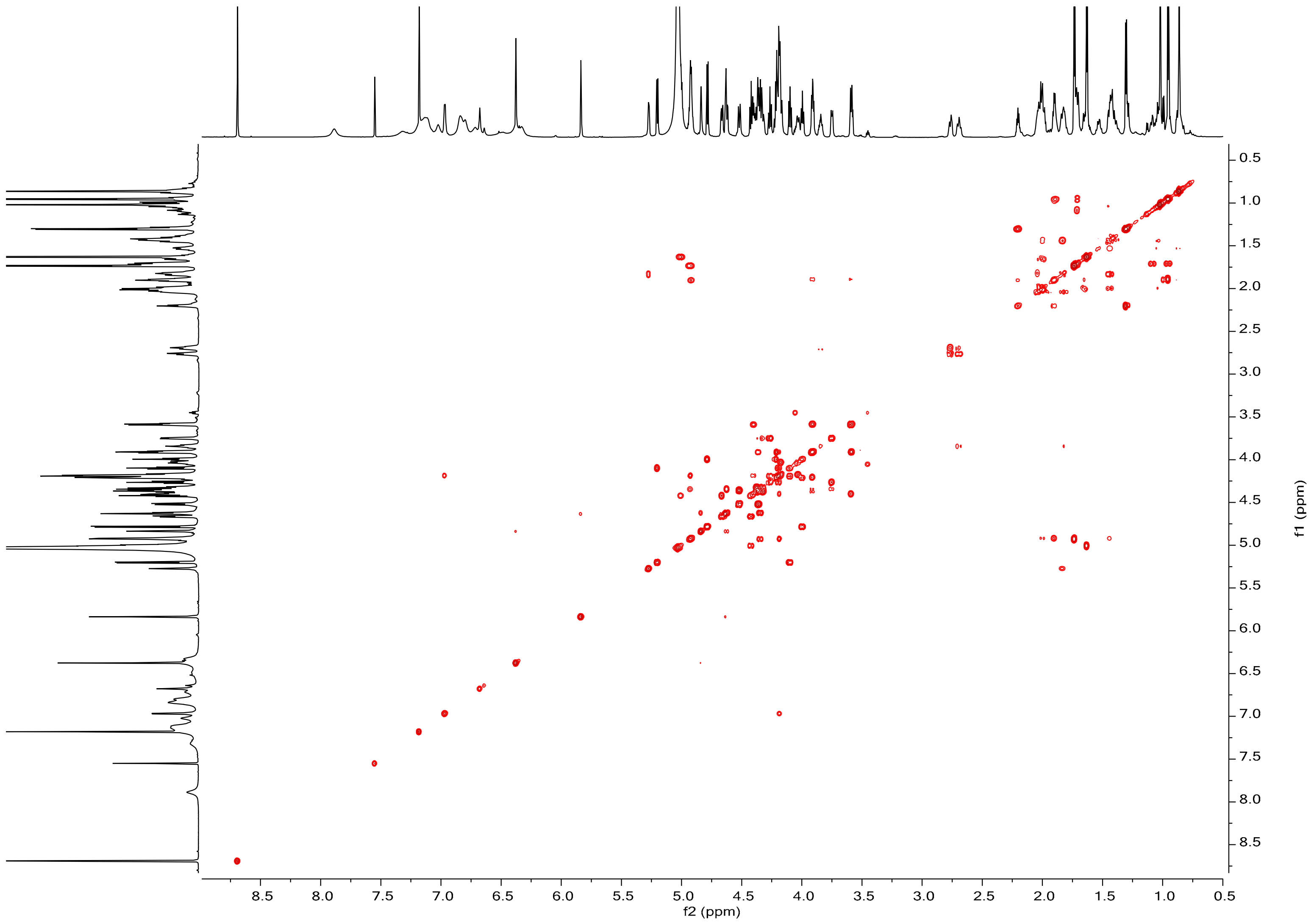

Figure S6. The HMBC spectrum of compound **1** in C_5D_5N

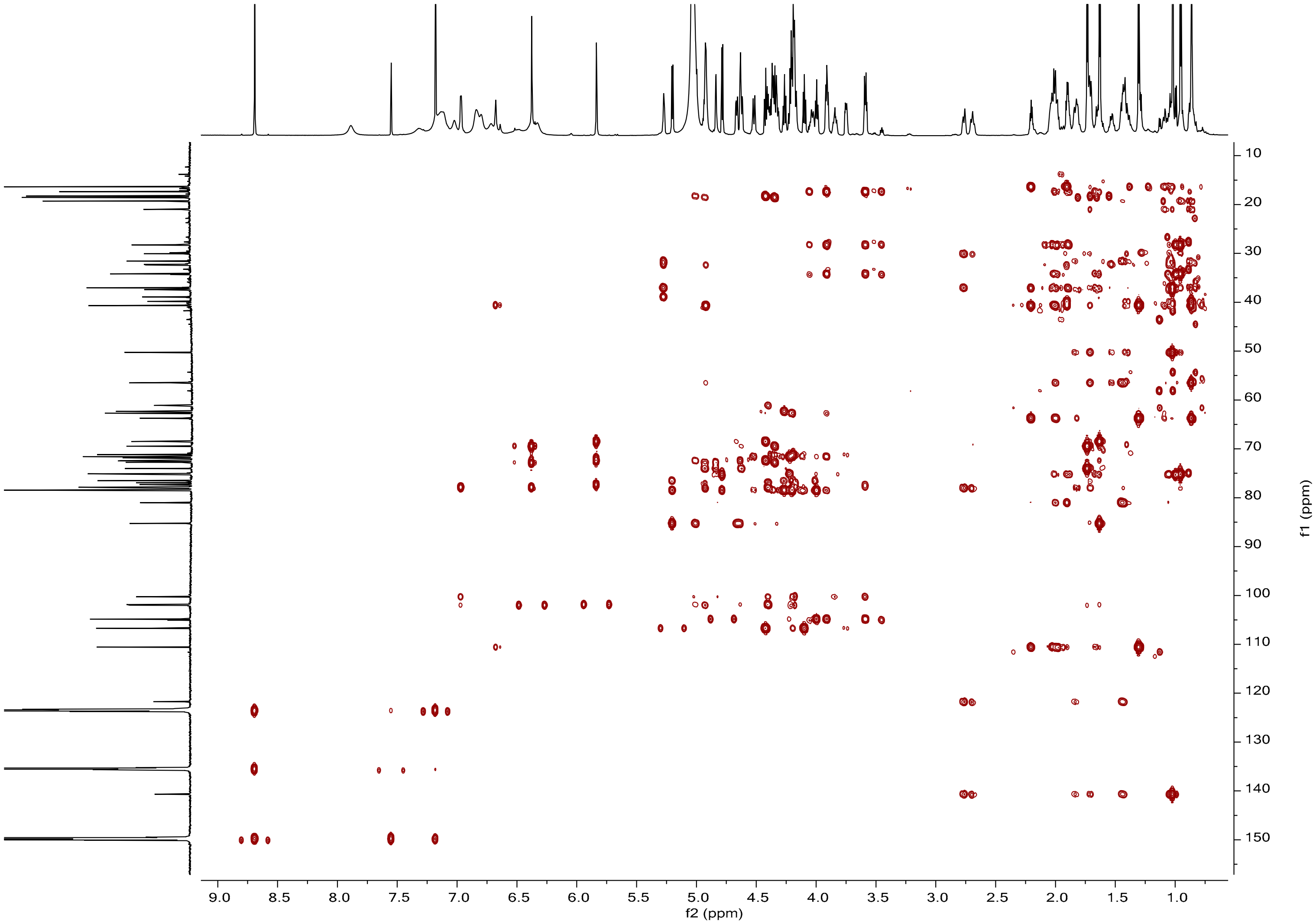

Figure S7. The NOESY spectrum of compound **1** in C_5D_5N

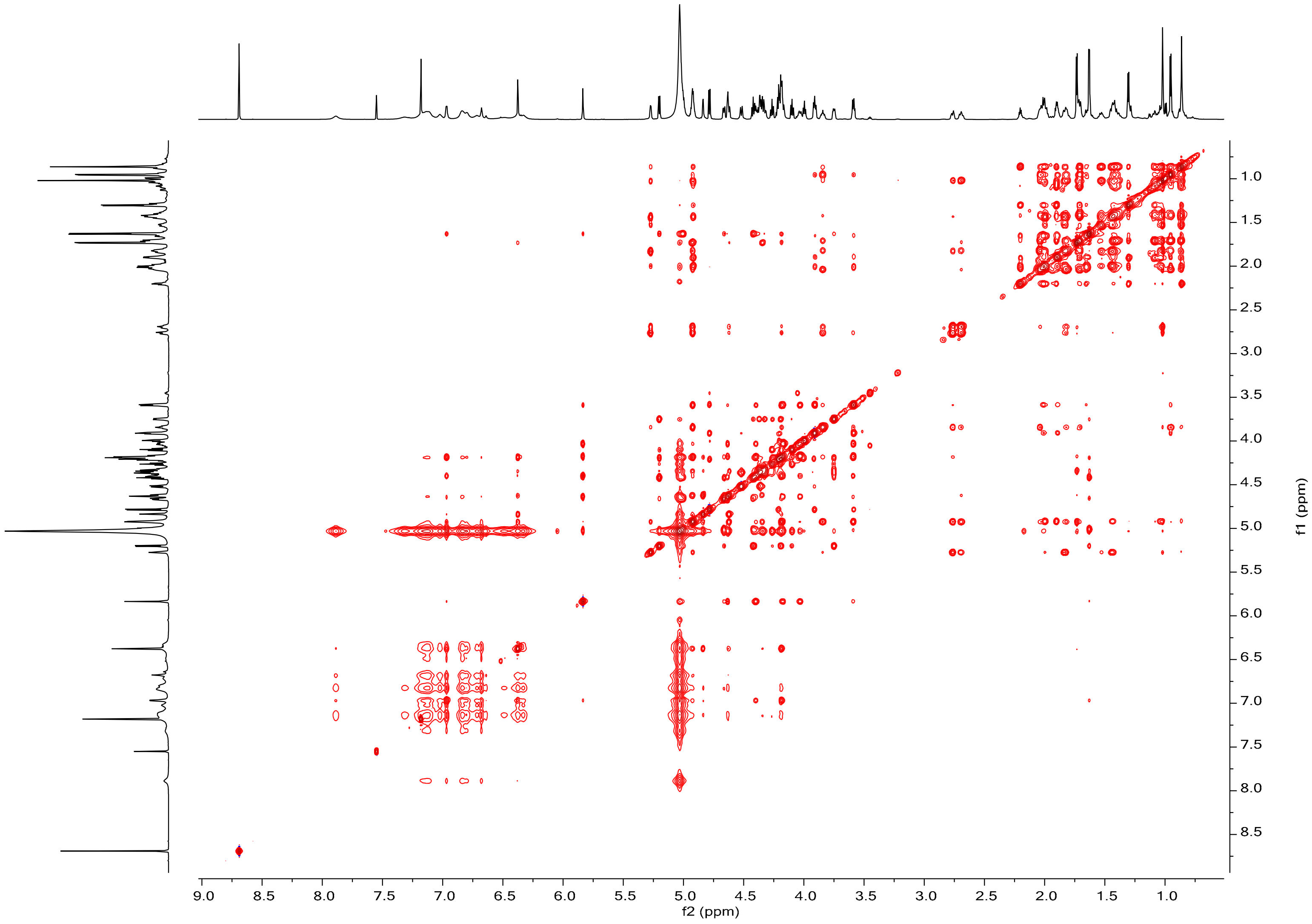

Supplement: Supplementary file 1 [file plants-10-02067-s001.zip › plants-1400403-supplementary.pdf]
